# Planar Cell Polarity Effector Proteins Inturned and Fuzzy Form a Rab23 GEF Complex

**DOI:** 10.1016/j.cub.2019.07.090

**Published:** 2019-10-07

**Authors:** Andreas Gerondopoulos, Helen Strutt, Nicola L. Stevenson, Tomoaki Sobajima, Tim P. Levine, David J. Stephens, David Strutt, Francis A. Barr

**Affiliations:** 1 Department of Biochemistry, University of Oxford, South Parks Road, Oxford OX1 3QU, UK; 2 Department of Biomedical Science, University of Sheffield, Firth Court, Sheffield S10 2TN, UK; 3 School of Biochemistry, University of Bristol, Biomedical Sciences Building, University Walk, Bristol BS8 1TD, UK; 4 Institute of Ophthalmology, University College London, 11-43 Bath St., London EC1V 9EL, UK

**Keywords:** cilia, primary cilium, planar polarity, Rab23, guanine nucleotide exchange factor, longin domain, C-plane, Inturned, Fuzzy, Carpenter syndrome

## Abstract

A subset of Rab GTPases have been implicated in cilium formation in cultured mammalian cells [1, 2, 3, 4, 5, 6]. Rab11 and Rab8, together with their GDP-GTP exchange factors (GEFs), TRAPP-II and Rabin8, promote recruitment of the ciliary vesicle to the mother centriole and its subsequent maturation, docking, and fusion with the cell surface [2, 3, 4, 5]. Rab23 has been linked to cilium formation and membrane trafficking at mature cilia [1, 7, 8]; however, the identity of the GEF pathway activating Rab23, a member of the Rab7 subfamily of Rabs, remains unclear. Longin-domain-containing complexes have been shown to act as GEFs for Rab7 subfamily GTPases [9, 10, 11, 12]. Here, we show that Inturned and Fuzzy, proteins previously implicated as planar cell polarity (PCP) effectors and in developmentally regulated cilium formation [13, 14], contain multiple longin domains characteristic of the Mon1-Ccz1 family of Rab7 GEFs and form a specific Rab23 GEF complex. In flies, loss of Rab23 function gave rise to defects in planar-polarized trichome formation consistent with this biochemical relationship. In cultured human and mouse cells, Inturned and Fuzzy localized to the basal body and proximal region of cilia, and cilium formation was compromised by depletion of either Inturned or Fuzzy. Cilium formation arrested after docking of the ciliary vesicle to the mother centriole but prior to axoneme elongation and fusion of the ciliary vesicle and plasma membrane. These findings extend the family of longin domain GEFs and define a molecular activity linking Rab23-regulated membrane traffic to cilia and planar cell polarity.

## Highlights

• Inturned and Fuzzy are tri-longin domain proteins
• Inturned and Fuzzy form a Rab23 GEF complex
• Rab23 GEF localizes to cilia and promotes cilium formation
• Rab23 acts downstream of Inturned and Fuzzy to regulate planar polarity in *Drosophila*

## Graphical Abstract

**Figure:**
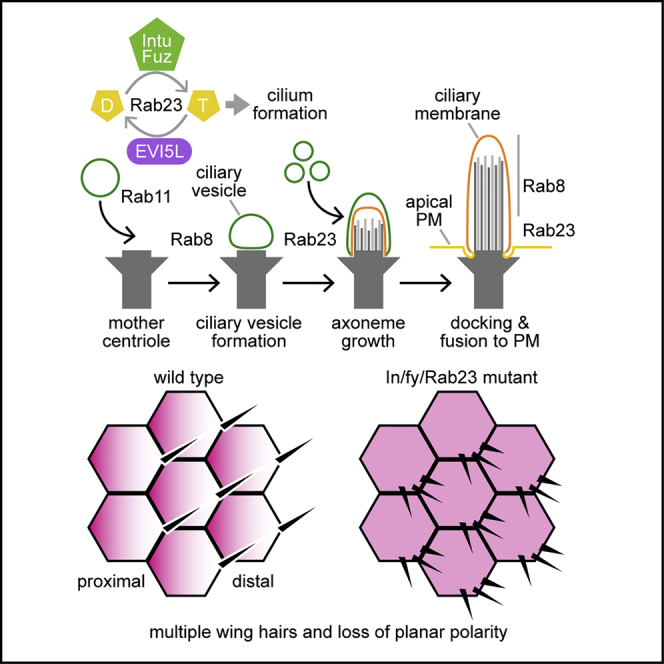

## Teaser

Gerondopoulos et al. show that the CPLANE components Inturned and Fuzzy form a conserved Rab23 GEF involved in the establishment of planar polarity and in cilium formation. Inturned and Fuzzy each contain 3 longin domains, with the first pair required for Rab23 GEF activity.

## Results

### Identification of the Rab23 GEF Complex

**Figure 1:**
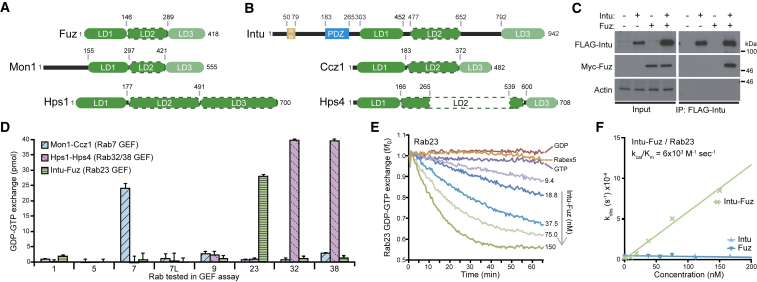
Inturned and Fuzzy Are Longin-Domain Proteins Related to Known Rab GEF Subunits and Form a Rab23 GEF Complex (A and B) Predicted domain structures of *H. sapiens* (A) Fuz, Mon1, and Hps1 and (B) Intu, Ccz1, and Hps4. Longin (green), PDZ (blue), and WW-like domains (gold) are shown. LD1, LD2, and LD3 indicate the positions of the predicted longin domains, many of which, in particular LD2 of Hps4, are extended beyond the minimal 120 residues by inserts in loops. All LD1s are canonical ββαβββαα longin domains (β, β sheet; α, α helix), all LD2s are αββαββββα circular permuted roadblock longin-type domains (dashed lines), and LD3s are typically ββαβββα lamtor-like longin domains, which lack the final helix (pale green), except for Hps1, where LD3 is of the roadblock type. (C) HEK293T cells were transfected with FLAG-Intu and Myc-Fuz as indicated. After 24 h, complexes were recovered using FLAG immunoprecipitation and western blotted for Intu and Fuz. Actin was used as a negative control. (D) GDP-GTP exchange endpoint assays were performed using human Intu-Fuz, Mon1-Ccz1, and Hps1-Hps4 complexes and a subset of Rab GTPases. Mean GDP-GTP exchange in pmol with error bars indicating the SEM for 3 independent experiments are plotted in the graph for each GEF complex. (E) GDP-GTP exchange activity of Intu-Fuzzy complexes toward Rab23 was measured over time as a function of GEF concentration. Rabex-5 was taken as a negative control. The basal exchange rate in the absence of a GEF was subtracted from the values plotted in the graph. (F) Initial rates of nucleotide exchange were extracted from these data for the Intu-Fuz complex or the individual subunits and plotted against GEF or subunit concentration for 3 independent experiments. Catalytic efficiency (k_cat_/K_M_) toward Rab23 was calculated as described in the STAR Methods. See also Figure S1.

Phylogenetic analysis shows that Rab23 is a member of the Rab7/32/38 family of Rabs acting in lysosome and lysosome-related organelle trafficking [15]. Rab7 family GTPases are activated by a conserved group of two-subunit GEF complexes, where each subunit is characterized by a single copy of a common α-β-α sandwich fold, the longin domain that is close to the N terminus [9, 10, 11, 12]. Longin domains are a defining feature of diverse GEFs and can therefore be used to identify these enzymes [16]. Because of the relationship between Rab23 and Rab7, we therefore searched for proteins related to the Mon1-Ccz1 Rab7 GEF complex using HHSearch [17, 18]. This approach revealed similarity not only with the Rab32/38 GEF BLOC-3 (subunits Hps1 and Hps4) but also with two other proteins, Inturned (Intu) and Fuzzy (Fuz), previously implicated as planar cell polarity effectors and in developmentally regulated cilium formation [14, 19]. Fuz shows homology over its entire length to both Mon1 and Hps1, and each of these three proteins is predicted to contain three longin domains (Figure 1A). Intu appears to have two protein interaction domains in its N terminus, a previously detected PDZ domain [20], and a region that we identify as being weakly similar to WW domains. Most important for this work, the C-terminal portion of Intu, after the PDZ domain, is homologous to the full length of Ccz1 and Hps4 (Figure 1B). Intu, Ccz1, and Hps4 are all predicted to contain three longin domains, in the same pattern as Fuz, Mon1, and Hps1 (Figure 1B). This indicates that Intu and Fuz are likely to have arisen by duplication of a single progenitor, already implicated as the ancestral gene for Ccz1, Mon1, Hps1, and Hps4 [12]. The similarity between the C-terminal half of Fuz and longin domains in vesicle coat proteins and the SNARE membrane fusion protein Ykt6 was noted previously [21]. However, at that time, it was not realized this was a feature of some Rab GEFs. Our prediction also adds to what was known about this entire family by identifying two hitherto overlooked longin-type domains in the region C-terminal to the known longin domain [16].

This pattern of homology indicates that Intu-Fuz are likely to form a functional pair, equivalent to Mon1-Ccz1 and Hps1-Hps4. Interaction mapping revealed that Intu and Fuz form a binary complex (Figures 1C and S1C). In part, this is likely to be mediated by the multiple longin domains in both proteins (Figures S1A and S1B). By analogy with the other longin domain containing Rab GEFs [11, 22], we propose that the multiple longin domains are likely to form sequential pairwise interactions.

In agreement with the proposed family relationship, Intu-Fuz complexes show specific GEF activity toward Rab23, but not other Rab7 subfamily GTPases, or Rab1, Rab5, and Rab9 (Figure 1D). This activity was comparable to the Rab7 and Rab32/38-specific GEFs Mon1-Ccz1 and Hps1-Hps4, respectively (Figure 1D). This activity required the first longin domain of Intu (Figure S1A), similar to mapping of Rab7 GEF activity to the first longin domains of Mon1-Ccz1 [11]. More detailed kinetic analysis confirmed that Rab23 is activated by Intu-Fuz in a concentration-dependent fashion, but not by the unrelated Rab5 GEF Rabex-5 (Figure 1E). Intu-Fuz has a high specific activity for Rab23, k_cat_/K_M_ ∼6 × 10^3^ M^−1^s^−1^ (Figure 1F), similar to other Rab GEFs [23]. Like other GEFs in this family, both subunits are required for specific GEF activity (Figure 1F). Finally, two disease-associated Intu mutants [24] resulted in reduced Rab23 GEF activity (Figure S1D). Based on these findings, we conclude that Intu-Fuz has the hallmarks of a specific Rab23 GEF.

### Rab23 GEF Localizes to Cilia and Promotes Cilium Formation

**Figure 2:**
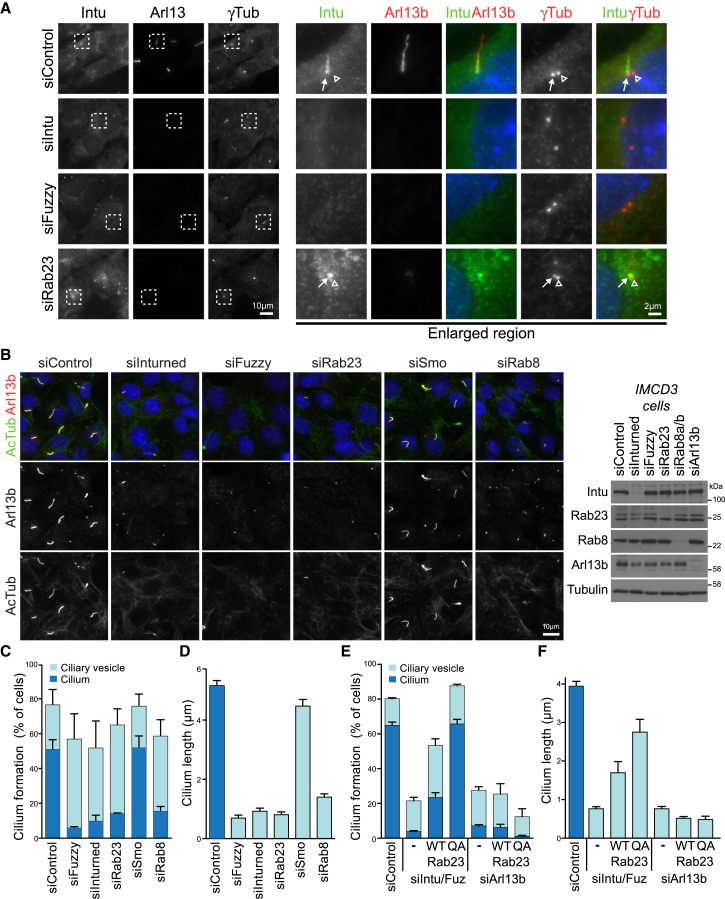
Inturned Is Associated with the Proximal Region of Cilia (A) IMCD3 cells depleted of Intu, Fuz, or Rab23 using small interfering RNA (siRNA) for 72 h were induced to form cilia by serum starvation for 14 h. The cells were fixed with TCA-glycine and then stained for Intu and the ciliary markers Arl13b and γ-tubulin. Enlarged panels show details of the cilium and basal bodies. Arrows mark Intu localization to the proximal region of the cilium and the γ-tubulin positive mother-daughter centriole pair; open arrowheads mark the position of the daughter centriole. (B) IMCD3 cells were depleted of Intu, Fuz, Rab23, Rab8, or Smoothened (Smo) for 72 h and then serum starved for 14 h. The cells were fixed with PFA and then stained with antibodies to Arl13b and acetylated tubulin (AcTub) or analyzed by western blotting to confirm depletion of target proteins. (C) Elongated cilia and punctate ciliary vesicles were identified using Arl13b staining (300 cells per condition in 4 independent experiments). Error bars indicate the SEM. (D) Cilium length (100 cells per condition in 3 independent experiments) was measured using Arl13b and acetylated tubulin. Errors bars indicate the SEM. (E) IMCD3 cells were depleted of Intu and Fuz or Arl13b for 48 h, mock transfected (−), or transfected with GFP-Rab23 (WT) or GFP-Rab23^Q68A^ (QA) for 24 h and then induced to form cilia for 14 h. The cells were fixed with PFA and then stained with antibodies to Arl13b and acetylated tubulin. Elongated cilia and punctate ciliary vesicles were identified using Arl13b staining (100 cells per condition in 3 independent experiments). Error bars indicate the SEM. (F) Cilium length (30 cells per condition in 2 independent experiments) was measured using Arl13b and acetylated tubulin. Errors bars indicate the SEM. See also Figures S2 and S3.

Previous work has shown that loss of Intu and Fuz in animal models disrupts ciliogenesis [14, 21, 25, 26]. Furthermore, Fuz has previously been shown to localize to centrioles and the basal region of cilia [27]. We therefore investigated the localization of endogenous Rab23 GEF complexes using Intu-specific antibodies. Comparison with two ciliary membrane markers, Arl13b and Rab8, revealed that Intu localizes to the basal region of the cilium adjacent to one of the centrioles (Figures 2A and S2A, arrows). This staining was lost in cells depleted of either Intu or Fuz, consistent with the biochemical data that these proteins function as a GEF complex. Rab23 depletion resulted in a collapse of the Intu staining to a small punctate structure overlapping one of the centrioles (Figure 2, arrows and arrowhead). Consistent with the idea that this is a ciliary vesicle precursor associated with the mother centriole, the structure was positive for Rab8 (Figure S2A, arrow). Similar results were obtained using the IFT-B component IFT88 as a marker for the interior of the cilium and ciliary vesicle (Figure S2B).

Loss and shortening of cilia to a single point, most likely to be a ciliary vesicle, was seen with depletion of Intu, Fuz, or Rab23 as well as Rab8 in IMCD3 cells (Figures 2C–2F). As a negative control, the ciliary membrane protein Smoothened, which is required for Hh signaling, but not cilium formation or maintenance, was depleted. As expected, the cilia were of the same length and observed at the same frequency as in control cells (Figures 2B–2D). These findings were confirmed in hTERT-RPE1 cells depleted of Intu, Fuz, or Rab23, which show fewer and shorter cilia (Figures S2C–S2F).

To provide additional support for the role of Rab23 at cilia, we investigated the role of its cognate GAP, EVI5L, which can be used to specifically inactivate Rab23 [1]. Similar to results obtained in hTERT-RPE1 cells [1], overexpression of catalytically active Rab23 GAP (EVI5L)—but not the catalytically inactive mutant—reduced cilium formation and cilium length in IMCD3 cells (Figures S3A and S3B). A structurally related GAP acting on Rab35 (EVI5) had no effect on cilium formation or length, and the Rab8 GAP (TBC1D30) reduced cilium formation and length as expected (Figures S3A and S3B).

Wild-type Rab23 localized to the plasma membrane in both hTERT-RPE1 and IMCD3 cells (Figures S3C and S3D). However, the slowly cycling Rab23^Q68A^ dominant active mutant was greatly enriched at the cilium (Figures S3C and S3D) but did not alter the length of cilia in either cell line (Figures S3E and S3F). This is different to the effects of Rab8 expression, which increases cilium length when overexpressed [1]. Notably, fewer than 20% of cells depleted of Intu-Fuz have cilia (Figure 2C), and those cilia that do form are shortened to <1 μm in length (Figure 2D).

We then asked whether overexpression of wild-type Rab23 or the dominant active Q68A mutant that accumulates in cilia could rescue the effects of Intu-Fuz depletion. Overexpression of wild-type Rab23 rescued cilium formation in over 50% of cells (Figure 2E), and cilium length increased to ∼2 μm (Figure 2F). The dominant active Rab23^Q68A^ mutant rescued cilium formation to the level seen in control cells (Figure 2E), and cilium length was increased to ∼3 μm compared to 4 μm in control cells (Figure 2F). To ascertain the specificity of these effects, Rab23 and Rab23^Q68A^ were expressed in cells depleted of Arl13b, which fail to assemble normal axonemes [28, 29]. In neither case could Rab23 overexpression rescue the effects of Arl13b depletion on cilium formation (Figures 2E and 2F).

Expression of dominant negative inactive Rab23^N121I^ and known disease-associated mutants Rab23^M12K, C85R^ [30, 31] resulted in reduced cilium formation (Figures S3D–S3F). Both Rab23^N121I^ and the two disease-associated mutants have reduced GDP-binding properties (Figure S3G) and show an increased rate of basal GTP-hydrolysis (Figure S3H). These mutants therefore rapidly bind, hydrolyze, and release nucleotide in the absence of any regulatory factors. This rapid cycling may explain why they act as dominant negative mutants, interfering with endogenous Rab23 function.

Taken together with published work [27], these results support the conclusion that Intu-Fuz complexes localize to the forming cilium and proximal region of mature cilia and function upstream of Rab23 to promote its activation.

### Rab23 Activation Is Required Downstream of Ciliary Vesicle Formation

**Figure 3:**
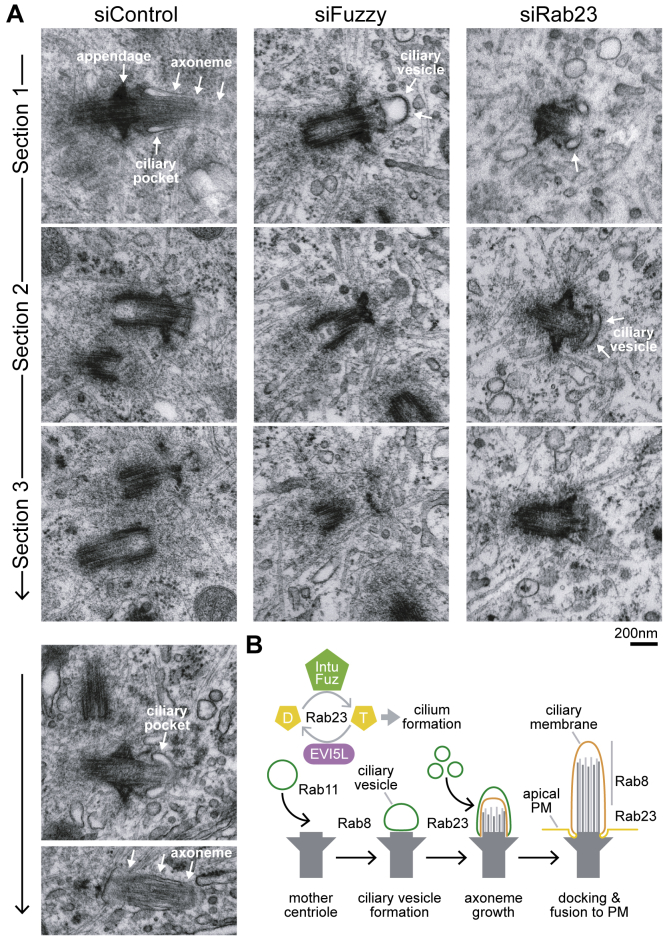
Rab23 Activation Is Required Downstream of Ciliary Vesicle Formation (A) hTERT-RPE1 cells were depleted of Fuzzy or Rab23 for 48 h and then induced to form cilia for a further 48 h. The cells were processed for serial section transmission electron microscopy and data collected for 10 control cells, 8 Fuzzy cells, and 13 Rab23-depleted cells. Serial sections enabled us to define the ciliary phenotype in 5 control, 4 Fuzzy cells, and 4 Rab23-depleted cells. Representative serial sections through the mother centriole and cilium are shown. Examples of axoneme structures in two control cells are shown. Arrows indicate the mother centriole appendages, ciliary pocket, and axoneme in control cells or the ciliary vesicle in Fuzzy or Rab23-depleted cells. (B) An updated model for the sequential action of Rab GTPases in cilium formation. Components of the Rab23 regulatory cycle are shown. Rab23 is activated by the Intu-Fuz GEF and inactivated by the EVI5L GAP. The ciliary vesicle and precursor membranes are depicted in green, the mature ciliary membrane in orange, and plasma membrane in yellow.

To further narrow down the stage at which Rab23 and the Rab23 GEF are required for cilium formation, hTERT-RPE1 cells were examined by transmission electron microscopy. Adjacent serial sections show the mother centriole marked by characteristic appendages, the associated axoneme, and ciliary pocket in control cells (Figure 3A). In cells depleted of either Fuz or Rab23, intact axonemes were not found, consistent with the loss of acetylated tubulin staining seen by light microscopy (Figures 2A and S2A). However, docked ciliary vesicles were observed by electron microscopy in both cases (Figure 3A), a phenotype seen previously in cells lacking Rab8 [5]. These observations support the idea that the Rab8-positive punctate structures seen by light microscopy in cells depleted of Rab23, Fuz, or Intu are ciliary vesicles docked to the mother centriole (Figure S2A). They also help us place Intu-Fuz and Rab23 downstream or parallel to Rab8 in the cilium formation pathway. Based on these findings, we propose that they function at an intermediate or late stage of cilium formation, after docking of the ciliary vesicle to the mother centriole but prior to axoneme elongation and fusion of the ciliary vesicle and plasma membrane (Figure 3B).

### Inturned and Fuzzy Act with Rab23 to Regulate Planar Polarized Trichome Formation in *Drosophila*

**Figure 4:**
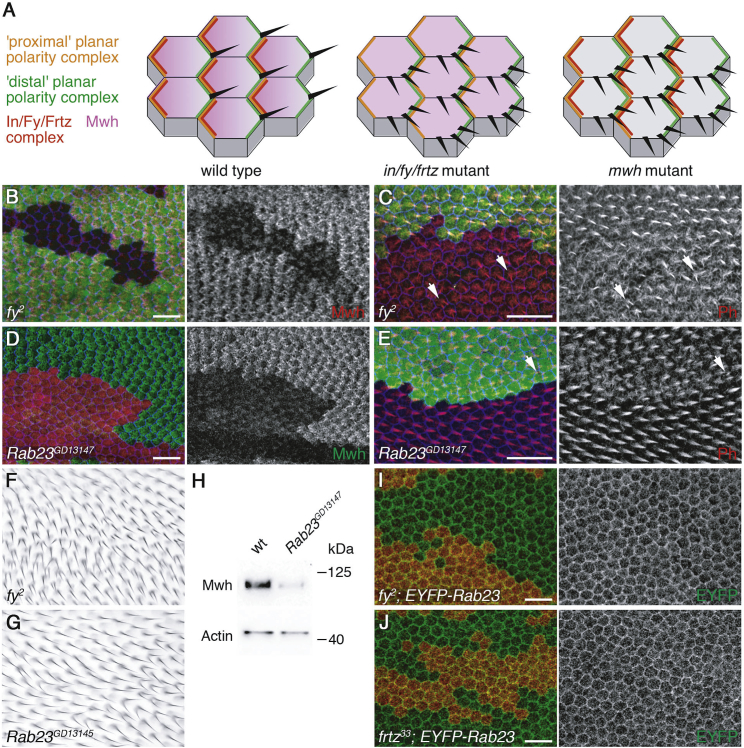
Rab23 Is Required for Planar Polarized Trichome Formation in the *Drosophila* Wing (A) Cartoons of *Drosophila* pupal wing cells show proximal and distal cellular localization of planar polarity proteins (orange and green, respectively), proximal localization of the putative In, Fy, and Frtz protein complex (red), and the proximal to distal gradient of apical Mwh localization (purple). Wild-type is left, showing production of a single distally pointing planar polarized trichome (black). Middle shows loss of In, Fy, or Frtz, where Mwh apical levels are low and not graded and multiple trichomes are produced that are not planar polarized relative to the planar polarity protein localization in the cell. Right shows loss of Mwh where In, Fy, and Frtz localization is normal but multiple non-planar polarized trichomes are produced. (B and C) Pupal wings carrying loss-of-function clones of *fy*^*2*^, marked by loss of GFP immunolabeling (green). (B) A 32 h after-puparium-formation (APF) wing, immunolabeled for Mwh (red) and Fmi (blue). (C) A pupal wing from a fly raised at 18°C for 64.5 h (32.5 h equivalent at 25°C), labeled for F-actin with phalloidin (red) and the junctional marker Armadillo (blue). Arrows indicate multiple trichomes emerging from the same cell. Note the general increase in the apical actin network in mutant cells. (D and E) Pupal wings with clones expressing RNAi against *Rab23*, marked by presence of β-gal immunolabeling (red in D and green in E). (D) A pupal wing from a fly raised at 29°C for 27 h, immunolabeled for Mwh (green) and Fmi (blue). (E) A pupal wing from a fly raised at 29°C for 27.5 h, labeled for F-actin with phalloidin (red) and Fmi (blue). Again, note the general increase in the apical actin network in mutant cells; arrows indicate multiple trichomes emerging from the same cell. (F and G) Adult wing of (F) *fy*^*2*^ fly or (G) wing from fly expressing RNAi against *Rab23* using the *ptc-GAL4* driver at 25°C. (H) Western blot of pupal wings from wild-type pupae or pupae expressing *Rab23* RNAi, raised at 29°C for 27 h. Blots probed with Mwh antibody or actin control are shown. (I and J) 31 h APF pupal wings expressing *EYFP-Rab23* and immunolabeled for GFP (green), carrying loss of function clones of *fy*^*2*^ (I) or *frtz*^*33*^ (J) marked by loss of β-gal labeling (red). All scale bars, 10 μm. See also Figure S4.

We then asked whether the regulation of Rab23 by Intu and Fuz is conserved. In the *Drosophila* pupal wing, In and Fy (the fly homologs of Intu and Fuz) are thought to act in a complex with the WD40-repeat protein Fritz (Frtz) (Wdpcp in vertebrates). In, Fy, and Frtz localize to proximal cell ends, where they regulate the phosphorylation and localization of the atypical formin multiple wing hairs (Mwh). Mwh then inhibits actin polymerization and restricts production of actin-rich trichomes to distal cell edges (Figures 4A–4C; reviewed in [32]). Interestingly, knockdown of *Drosophila Rab23* using RNAi caused a trichome duplication phenotype in the adult wing, as seen in *fy*, *in*, and *frtz* mutants (Figures 4F and 4G) [33]. We therefore investigated whether Rab23 acts together with Fy, In, and Frtz to regulate Mwh localization. Strikingly, loss of Rab23 activity in the pupal wing resulted in a loss of apical Mwh localization and excess actin polymerization, thus phenocopying *fy* mutants (Figures 4C–4E and S4A). Furthermore, loss of Rab23 resulted in a decrease in phosphorylation and overall cellular levels of Mwh (Figure 4H). Notably, loss of Frtz causes a decrease in Mwh phosphorylation, but Mwh levels are normal [34]. Loss of Rab23 activity did not affect the junctional localization of Frtz or Fy, as expected (Figures S4B and S4C). Furthermore, the plasma membrane association of GFP-tagged Rab23 [33, 35] is not affected by loss of In or Fy (Figures 4I and 4J), suggesting that Rab23 activity, but not its localization, is regulated by In, Fy, and Frtz. These findings are consistent with the idea that Rab23 functions in a conserved pathway downstream of Intu-Fuz.

## Discussion

### Intu-Fuz Expand the Family of Longin Domain Rab GEFs

Intu, Fuz, and the WD40 repeat protein WDPCP/Fritz form part of the ciliogenesis and planar polarity effector protein complex (CPLANE) [24]. Here, we show that the Intu-Fuz subcomplex of CPLANE has GEF activity toward Rab23. Consistent with this biochemical activity, we find that Rab23 functions downstream of Intu-Fuz in cilium formation in mammalian cultured cells and planar polarized trichome localization in *Drosophila*. The existing Rab7 family GEFs, Mon1-Ccz1 and Hps1-Hps4, are known to have two longin domains, forming the enzymatic core of the GEF. Our analysis shows that these GEFs and Intu-Fuz have 6 longin domains, likely to form 3 dimeric platforms. We suggest that these complexes are named tri-longin domain Rab (TLDR) GEFs to distinguish them from complexes such as the Rab1 GEF TRAPP, which have a dimeric longin domain catalytic site or single longin domain DENN-family Rab GEFs [22, 36]. The known TLDR GEFs Mon1-Ccz1 and Hps1-Hps4 activate Rab7 and Rab32/38 in pathways directing traffic from early endosomal compartments to lysosomes and lysosome-related organelles, respectively [9, 10, 11]. Endocytic trafficking is crucial for the regulation and formation of cilia [37], and the evolutionary relationship with Rab7 and Mon1-Ccz1 suggests that Rab23 and Intu-Fuz are conserved components governing trafficking between endosomal and ciliary compartments.

In addition to contributing to Rab23 GEF activity, Fuz interacts with the Ras superfamily GTPase RSG1, also present at the base of cilia and required for a late stage of cilium formation [21, 38, 39]. RSG1 is strongly associated with the CPLANE through Fuz [21, 24]. This tight association is unusual for GTPase-GEF interactions, since these are typically transient. One interesting possibility is that the local recruitment or activity of Intu-Fuz toward Rab23 is mediated through interactions with RSG1. Since longin and related roadblock domains have been proposed to be versatile binding platforms for GTPases [12, 16], the presence of multiple such domains in Intu-Fuz would be consistent with interaction with more than one GTPase. This is an area requiring further investigation.

### Intu-Fuz and Rab23 Activation in Planar Polarity and at Cilia during Development

Rab23 is mutated in Carpenter syndrome, an autosomal recessive human developmental disorder characterized by open neural tube and craniofacial defects [30, 31]. Similarly, recessive Rab23 nonsense mutations in the *open brain* mouse are embryonically lethal due to open neural tube defects and other changes reminiscent of Carpenter syndrome [40, 41]. Mouse developmental studies indicate Rab23 loss of function results in defective left-right patterning and dysregulated nodal and Hh signaling, both pathways associated with cilia [41, 42, 43]. Despite these defects, nodal cilia were found to be morphologically normal in one of these studies [42]. Previous reports and the work shown here indicate that cilium formation in some cultured cell lines is perturbed following depletion of Rab23 or suppression of Rab23 function through removal of the GEF or overexpression of the GAP [1]. Rab23 is also required for trafficking of the Hh signaling regulator Smoothened and dopamine receptors to mature cilia in a pathway linked to the kinesin Kif17 and the intraflagellar transport machinery [7, 8]. Therefore, although Rab23 may not be essential for the formation of all cilia during development, it plays an important role in membrane trafficking at cilia.

While many questions remain about the interplay between multiple membrane trafficking events during cilium formation, the mechanistic link established in this work between Intu-Fuz and Rab23 activation will be valuable for informing future studies of cilia and the establishment of planar polarity during development.

## STAR★Methods

### Key Resources Table

**Table:** 

REAGENT or RESOURCE	SOURCE	IDENTIFIER
**Antibodies**		

Mouse monoclonal α-tubulin DM1A	Sigma-Aldrich	Cat# T6199; RRID:AB_477583
Mouse monoclonal actin clone AC-74	Sigma-Aldrich	Cat# A5316; RRID:AB_476743
Mouse monoclonal FLAG-epitope M2	Sigma-Aldrich	Cat# F3165; RRID:AB_259529
Rabbit polyclonal FLAG-epitope F7425	Sigma-Aldrich	Cat# F7425; RRID:AB_439687
Rabbit polyclonal Arl13b	Proteintech	Cat# 17711-1-AP; RRID:AB_2060867
Monoclonal c-MYC	Developmental Studies Hybridoma Bank	Cat# 9E 10; RRID:AB_2266850
Mouse monoclonal acetylated-tubulin clone 6-11B-1	Sigma-Aldrich	Cat# T7451; RRID:AB_609894
Mouse monoclonal γ-tubulin T6557	Sigma-Aldrich	Cat# T6557; RRID:AB_477584
Rabbit monoclonal Rab8A (D22D8) XP	Cell Signaling Technology	Cat# 6975; RRID:AB_10827742
Goat polyclonal IFT88	Abcam	Cat# ab42497, RRID:AB_778681
Rabbit polyclonal Rab23	Proteintech	Cat# 11101-1-AP; RRID:AB_2173784
Mouse monoclonal Rab23	Proteintech	Cat# 60056-1-Ig; RRID:AB_2173782
Sheep polyclonal to aa1-1271 of human Inturned	This paper	Sheep α-hsIntu
Peroxidase-AffiniPure Donkey Anti-Rabbit IgG (H+L)	Jackson ImmunoResearch	Cat# 711-035-152; RRID:AB_10015282
Peroxidase-AffiniPure Donkey Anti-Mouse IgG (H+L)	Jackson ImmunoResearch	Cat# 715-035-151; RRID:AB_2340771
Peroxidase-AffiniPure Donkey Anti-Sheep IgG (H+L)	Jackson ImmunoResearch	Cat# 713-035-147; RRID:AB_2340710
Donkey anti-Rabbit IgG (H+L) Highly Cross-Adsorbed Secondary Antibody, Alexa Fluor 555	Thermo Fisher Scientific	Cat# A-31572; RRID:AB_162543
Donkey anti-Mouse IgG (H+L) Highly Cross-Adsorbed Secondary Antibody, Alexa Fluor 488	Thermo Fisher Scientific	Cat# A-21202; RRID:AB_141607
Donkey anti-Mouse IgG (H+L) Highly Cross-Adsorbed Secondary Antibody, Alexa Fluor 647	Thermo Fisher Scientific	Cat# A-31571; RRID:AB_162542
Donkey anti-Sheep IgG (H+L) Cross-Adsorbed Secondary Antibody, Alexa Fluor 488	Thermo Fisher Scientific	Cat# A-11015; RRID:AB_2534082
Mouse monoclonal Flamingo (Fmi)	Developmental Studies Hybridoma Bank	Cat# Flamingo 74; RRID:AB_528247
Mouse monoclonal Armadillo (Arm)	Developmental Studies Hybridoma Bank	Cat# N2 7A1 ARMADILLO; RRID:AB_528089
Rabbit polyclonal GFP	Abcam	Cat# ab6556; RRID:AB_305564
Rabbit polyclonal β-gal	ICN Pharmaceuticals	Cat# 55976; RRID:AB_2313707
Mouse monoclonal β-gal	Promega	Cat# Z3783; RRID:AB_430878
Rat Mwh	[34]	Rat α-Mwh
Rabbit Mwh	[34]	Rabbit α-Mwh
Rabbit Frtz	[34]	Rabbit α-Fritz
Mouse monoclonal AC-40 Actin	Sigma-Aldrich	Cat# A4700; RRID:AB_476730
Alexa Fluor 568 Phalloidin	Thermo Fisher Scientific	Cat# A12380

**Bacterial and Virus Strains**		

XL1-Blue Competent Cells	Agilent Technologies	Cat# 200249
BL21-CodonPlus (DE3)-RIL Competent Cells	Agilent Technologies	Cat# 230245

**Chemicals, Peptides, and Recombinant Proteins**		

Dulbecco’s modified Eagle’s medium	Thermo Fisher Scientific	Cat# 31966-047
Fetal Bovine Serum	Sigma-Aldrich	Cat# F9665
Bovine Calf Serum	Thermo Fisher Scientific	Cat# 16030074
GlutaMAX Supplement	Thermo Fisher Scientific	Cat# 35050061
Dulbecco’s Modified Eagle’s Medium/Nutrient Mixture F-12 Ham	Sigma-Aldrich	Cat# D6421
TrypLE Express Enzyme	Thermo Fisher Scientific	Cat# 12605036
Opti-MEM	Thermo Fisher Scientific	Cat# 11058021
Mirus TransIT-X2	Mirus Bio LLC	Cat# MIR 6000
Mirus LT1	Mirus Bio LLC	Cat# MIR 2306
Oligofectamine	Thermo Fisher Scientific	Cat# 12252011
Trichloracetic acid	Sigma-Aldrich	Cat# T6399
Paraformaldehyde	Sigma-Aldrich	Cat# 1581127
Moviol 4-88	Millipore	Cat# 475904
Glutaraldehyde	Sigma-Aldrich	Cat# G5882
Sodium cacodylate buffer pH 7.4	Molecular Dimensions	Cat# MD2-021-7.4
Osmium tetroxide	Agar Scientific	Cat# R1024
TAAB 812 resin	TAAB Laboratories Equipment Ltd	Cat# T026
Uranyl acetate	BDH	Cat# 10288
Lead nitrate	Sigma-Aldrich	Cat# L6258
Tri-Sodium citrate	BDH	Cat# 10242
Enhanced chemiluminescence (ECL) reagent	GE Healthcare	Cat# RPN2106
Anti-FLAG M2 affinity gel	Sigma-Aldrich	Cat# A2220
FLAG-peptide	Sigma-Aldrich	Cat# F3290
Ni-NTA Agarose	QIAGEN	Cat# 30230
Glutathione Sepharose 4B Media	GE Healthcare	Cat# 17-0756-05
Bovine Serum Albumin (IgG-Free, Protease-Free)	Jackson ImmunoResearch	Cat# 001-000-161
Activated charcoal	Sigma-Aldrich	Cat# C5510
[^3^H]-GDP (10 mCi/ml; 5000 Ci/mmol)	Hartmann Analytic	Cat# ART1736
γ-[^32^P]GTP (10 mCi/ml; 5,000 Ci/mmol)	Hartmann Analytic	Cat# FP-402
2′-(3′)-bis-O-(N-methylanthraniloyl)-GDP (Mant-GDP)	Jena Bioscience	Cat# NU-204
Guanosine 5′-triphosphate sodium salt hydrate	Sigma-Aldrich	Cat# G8877
Guanosine 5′-diphosphate sodium salt	Sigma-Aldrich	Cat# G7127
Ultima gold (liquid scintillation cocktail)	PerkinElmer	Cat# 6013329
Normal goat serum	Thermo Fisher Scientific	Cat# 16210064
1,4-diazabicyclo[2.2.2]octane (DABCO)	Sigma-Aldrich	Cat# D27802
Methyl salicylate	Sigma-Aldrich	Cat# M6572
Canada Balsam	Sigma-Aldrich	Cat# C1795

**Experimental Models: Cell Lines**		

mIMCD-3	ATCC	ATCC Cat# CRL-2123; RRID:CVCL_0429
hTERT RPE-1	ATCC	ATCC Cat# CRL-4000; RRID:CVCL_4388
HEK293T	ATCC	ATCC Cat# CRL-11268; RRID:CVCL_1926

**Experimental Models: Organisms/Strains**		

*D. melanogaster*: *y w Ubx-FLP; fy*^*2*^*FRT40/ubn-GFP FRT40*	This study	N/A
*D. melanogaster*: *Rab23*^*GD13147*^*; Actin > y+ > GAL4, UAS-lacZ/+; UAS-Dcr2/+*	This study	N/A
*D. melanogaster*: *w; ptc-GAL4/Rab23*^*GD13145*^*; UAS-Dcr2/+*	This study	N/A
*D. melanogaster*: *w* and *w Rab23*^*GD13147*^*/w; Actin-GAL4, tub-GAL80*^*ts*^*/+*	This study	N/A
*D. melanogaster*: *y w Ubx-FLP; fy*^*2*^*FRT40/arm-lacZ FRT40; EYFP-Rab23 /+*	This study	N/A
*D. melanogaster*: *y w Ubx-FLP; frtz*^*33*^*FRT40/arm-lacZ FRT40; EYFP-Rab23/+*	This study	N/A
*D. melanogaster*: *y w Ubx-FLP; FRT82 Rab23*^*T69A*^*/FRT82 arm-lacZ*	This study	N/A
*D. melanogaster*: *Rab23*^*GD13147*^*; Actin > y+ > GAL4, UAS-lacZ/+; UAS-Dcr2/+*	This study	N/A
*D. melanogaster*: *y w Ubx-FLP; Actin > EGFP-Fy FRT82 Rab23*^*T69A*^*/FRT82 arm-lacZ*	This study	N/A

**Oligonucleotides**		

siRNA targeting mouse Rab23, CAAGAAAACCAUCGGC GUA, UCGUACAACCAUUGCGUAU, UAUCAGGAACGAU CGGUAA, AUGACUAACUACAUCGGUA	Dharmacon	Cat# L-040868-01
siRNA targeting mouse Inturned UCACUAUAGUACUCG UUAA, CGAAGCAGGCAGACGGAGA	Dharmacon	Cat# J-066771-05, J-066771-07
siRNA targeting mouse Fuzzy CCAGCUGGACCCACAG UUA, AAACAAGAGGACACAGUCU	Dharmacon	Cat# J-058818-11, J-058818-12
siRNA targeting mouse Arl13b AGGACCAGUUCUUGCG AAU, GGGCUGAACGAGUCCGGAA, AGAGCAUCCUGA AGACGUA, UGGAGAAGCUGGUCAACGA	Dharmacon	Cat# L-042588-01
siRNA targeting mouse Rab8a CAGGAGCGGUUUCGAA CAA, GUAUCAUGCUGGUCUACGA, CAGAAGGUAGCC AGCGGUA, CGGACUCGAUUGAGAAAUU	Dharmacon	Cat# L-055301-01
siRNAs targeting mouse Rab8b CGAUAGAACUCGACGG AAA, CGAACAAUUACGACAGCAU, GCGUAAUCUUAGA CUCUUA, GGACAAAUUAGGCAGACUU	Dharmacon	Cat# L-040860-01
siRNAs targeting mouse Smo CAAUUGGCCUGGUGCU UAU, GAGCGUAGCUUCCGGGACU, GGAGUAGUCUGG UUCGUGG, GCUACAAGAACUAUCGGUA	Dharmacon	Cat# L-041026-00
siRNA targeting human Inturned ACAGAUAGCUUGACCA CUU, GGGUUAACCUUGUAGCUGU	Dharmacon	Cat# J-031873-09, J-031873-11
siRNA targeting human Fuzzy GCGAGGACCGAGAACA CGA, GUGUGUGGACUGCGUGAUU	Dharmacon	Cat# J-016342-11, J-016342-19
siRNA targeting human Rab23 GAACUAACGCAUUCAA GUA, CAAGUAUGAUUCAGCGAUA, CUGGAUGAUUCU UGUAUAA, GAUGGUGGUUGUAGGGAAU	Dharmacon	Cat# L-009789-00
siRNAs targeting human Rab8a CAGGMCGGUUUCGGA CGA, GAAUUAAACUGCAGAUAUG, GAACMGUGUGAU GUGAAU, GAAUUAAACUGCAGAUAUG	Dharmacon	Cat# L-003905-00
siRNAs targeting human Rab8b GCAAUUGACUAUGGGA UUA, GAACAAUCACGACAGCGUA, GAUCAAAGAAGAC CAGUUU, CGAUAGAACUAGAUGGAAA	Dharmacon	Cat# L-008744-00
Luciferase GL2 Duplex (siControl)	Dharmacon	Cat# D-001100-01

**Recombinant DNA**		

pcDNA5/FRT/TO/Myc /Fuzzy 1-146aa	This paper	pFB8869
pcDNA5/FRT/TO/Myc /Fuzzy 147-285aa	This paper	pFB8870
pcDNA5/FRT/TO/Myc /Fuzzy 147-485aa	This paper	pFB8871
pcDNA5/FRT/TO/Myc /Fuzzy 1-286aa	This paper	pFB8872
pcDNA5/FRT/TO/Myc /Fuzzy 287-483aa	This paper	pFB8873
pcDNA5/FRT/TO/Flag Intu 1-271aa	This paper	pFB7756
pcDNA5/FRT/TO/Flag Intu 1-450aa	This paper	pFB7758
pcDNA5/FRT/TO/Flag Intu 272-450aa	This paper	pFB7759
pcDNA5/FRT/TO/Flag Intu 272-942aa	This paper	pFB7760
pcDNA5/FRT/TO/Flag Intu 272-750aa	This paper	pFB7761
pcDNA5/FRT/TO/Flag Intu 451-942aa	This paper	pFB7762
pcDNA5/FRT/TO/Flag Intu 1-750aa	This paper	pFB7763
pcDNA5/FRT/TO/Flag Intu 176-942aa	This paper	pFB7747
pcDNA5/FRT/TO/Flag Intu 1-175aa	This paper	pFB7765
pcDNA5/FRT/TO/Flag Intu	This paper	pFB6514
pcDNA5/FRT/TO/Myc Fuzzy	This paper	pFB7095
pcDNA5/FRT/TO/Flag Fuzzy	This paper	pFB7094
pcDNA5/FRT/TO/GFP Rab23 M12K	This paper	pFB8640
pcDNA5/FRT/TO/GFP Rab23 C85R	This paper	pFB8637
pcDNA5/FRT/TO/GFP Rab23	This paper	pFB8863
pcDNA5/FRT/TO/GFP Rab23 Q68A	This paper	pFB4083
pcDNA5/FRT/TO/GFP Rab23 N121I	This paper	pFB4899
pEGP-C2/EVI5L WT	[1]	pFB3812
pEGP-C2/EVI5L RA	[1]	pFB3816
pEGP-C2/EVI5 WT	[1]	pFB3732
pEGP-C2/EVI5 RA	[1]	pFB3733
pEGP-C2/TBC1D30 WT	[1]	pFB4420
pEGP-C2/TBC1D30 RA	[1]	pFB4434
pcDNA5/FRT/TO/Flag Intu A452T	This paper	pFB9522
pcDNA5/FRT/TO/Flag Intu E500A	This paper	pFB9523
pcDNA5/FRT/TO/Myc Hps1	[10]	pFB6521
pcDNA5/FRT/TO/Flag Hps4	[10]	pFB6517
pcDNA5/FRT/TO/Flag Mon1a	[10]	pFB6180
pcDNA5/FRT/TO/Myc Ccz1	[10]	pFB6506
pET14-ccdB Intu 1-271aa	This paper	pFB7705
pFAT2-Rab1a	[1]	pFB3174
pFAT2-Rab5	[1]	pFB3090
pFAT2-Rab7	[1]	pFB3558
pFAT2-Rab7-like	[1]	pFB2116
pFAT2-Rab9a	[1]	pFB3179
pFAT2-Rab23	[1]	pFB3184
pFAT2-Rab32	[1]	pFB4624
pFAT2-Rab38	[1]	pFB4626
pQE32 TEV Rabex-5	[23]	pFB4981

**Software and Algorithms**		

Metamorph 7.5	Molecular Dynamics Inc	https://www.moleculardevices.com
MicroWin 2000 4.41	Berthold Technologies	https://www.berthold.com
Fiji 2.0.0-rc-49/1.52i	NIH Image	http://fiji.sc/
Prism 5.0	GraphPad Software	https://www.graphpad.com
Adobe Illustrator CS3	Adobe Systems Inc	https://www.adobe.com
Adobe Photoshop CS3	Adobe Systems Inc	https://www.adobe.com

### Lead Contact and Materials Availability

Further information and request for resources and reagents, such as plasmids, antibodies and fly strains generated in this study should be directed to and will be fulfilled by the lead contact, Francis Barr (francis.barr@bioch.ox.ac.uk).

### Experimental Model and Subject Details

#### Mammalian cell lines

HEK293T cells were cultured at 37°C and 5% CO_2_ in DMEM containing 10% [vol/vol] fetal bovine serum (Invitrogen). IMCD3 and hTERT-RPE1 cells were cultured at 37°C and 5% CO_2_ in a 1:1 mixture of DMEM and HAMS F12, 10% [vol/vol] bovine calf serum (Thermo Fisher Scientific) supplemented with 2.5 mM Glutamax™, and 1.2g/l sodium bicarbonate. For passaging, cells were washed in PBS, and then removed from the dish by incubation with TripLE Express (Thermo Fisher Scientific).

#### E.coli strains

BL21(DE3) were grown in LB and induced at 37°C overnight for expression of recombinant Rab proteins.

#### Fly strains

*Drosophila melanogaster* flies were grown on standard cornmeal/agar/molasses media at 18°C or 25°C, in plastic vials in a controlled humidity environment, on a 12 hr/12 hr light-dark cycle. For pupal wing dissections, pupae were aged for 28 hr after puparium formation (APF) at 25°C, or for 32 hr APF for trichome staining.

Fly strains are described in the Key Resources Table. *fy*^*2*^ and *frtz*^*33*^ are null mutations and *Rab23*^*T69A*^ carries a point mutation in the GTPase domain of *Rab23*. RNAi lines *Rab23*^*GD13145*^ and *Rab23*^*GD13147*^ were obtained from the Vienna *Drosophila* Resource Centre (VDRC). *EYFP-Rab23* is a knock-in of EYFP into the endogenous locus of Rab23 [35], and *EGFP-Fy* was expressed under the *Actin5C* promoter [34].

#### Genotypes of experimental models

The full genotypes used in each figure are listed below:

Figure 4

(B, C) y w Ubx-FLP; fy^2^ FRT40/ubn-GFP FRT40
(D, E) Rab23^GD13147^; Actin > y+ > GAL4, UAS-lacZ/+; UAS-Dcr2/+
(F) fy^2^
(G) w; ptc-GAL4/Rab23^GD13145^; UAS-Dcr2/+
(H) w and w Rab23^GD13147^/w; Actin-GAL4, tub-GAL80^ts^/+
(I) y w Ubx-FLP; fy^2^ FRT40/arm-lacZ FRT40; EYFP-Rab23 /+
(J) y w Ubx-FLP; frtz^33^ FRT40/arm-lacZ FRT40; EYFP-Rab23/+

Figure S4

(A) y w Ubx-FLP; FRT82 Rab23^T69A^/FRT82 arm-lacZ
(B) Rab23^GD13147^; Actin > y+ > GAL4, UAS-lacZ/+; UAS-Dcr2/+
(C) y w Ubx-FLP; Actin > EGFP-Fy FRT82 Rab23^T69A^/FRT82 arm-lacZ

### Method Details

#### Molecular Biology and Rab protein expression

Human Rab GTPases, Rab GAPs, Rabex-5, Mon1, Ccz1, Hps1 and Hps4 were previously amplified using PCR from human testis, fetus, and liver cDNA and cloned into pFAT2 for bacterial expression or pGFP-C2 for eukaryotic expression of GFP-tagged Rabs [10]. Inactive Rab GAP mutants EVI5^R208A^, EVI5L^R160A^ and TBC1D30^R349A^ have been characterized previously [1]. Human Inturned and Fuzzy were amplified by PCR from human testis cDNA. Point mutations were introduced using the Quickchange method. Mammalian expression constructs were made using pcDNA4/TO and pcDNA5/FRT/TO vectors (Invitrogen). Rab proteins in pFAT2 were expressed in BL21 (DE3) pRIL at 18°C for 12-14h. Cell pellets were disrupted in 20ml IMAC20 (20mM Tris-HCl, pH 8.0, 300mM NaCl, 20mM imidazole, and protease inhibitor cocktail; Roche) using an Emulsiflex C-5 system (Avestin Inc.). Lysates were clarified by centrifugation at 16,000 rpm in a JA-17 rotor for 30min. To purify the tagged protein, 0.5ml of nickel-charged NTA-agarose (QIAGEN) was added to the clarified lysate and rotated for 2h. The agarose was washed three times with IMAC20 and the bound proteins eluted in IMAC200 (IMAC20 with 200mM imidazole) collecting 1.5ml fractions. All manipulations were performed on ice or in an 8°C cold room. Purified proteins were dialyzed against TBS (50mM Tris-HCl, pH 7.4, and 150mM NaCl) and then snap frozen in liquid nitrogen for storage at −80°C. Protein concentration was measured using the Bradford assay.

#### Protein interaction mapping

For protein interaction mapping, FLAG- and myc-tagged full-length or truncation mutants of Inturned and Fuzzy were co-expressed in 1x 10cm dish of 70% confluent HEK293T cells. For this purpose, 400μl OptiMEM (ThermoFisher Scientific) was mixed with 14μl Mirus LT1 (Mirus Bio LLC), and after 5min 2μg of each plasmid DNA was added. After 25min this transfection mix was added to the cells. After 24h growth the cell pellet was lysed for 30min on ice in 500μl cell lysis buffer (50mM Tris-HCl, pH7.4, 150mM NaCl, 1% [vol/vol] NP-40, 0.1% [wt/vol] sodium deoxycholate and protease inhibitor cocktail). Cell extracts were clarified by centrifugation at 20,000xg in Eppendorf 5417R microfuge for 30 min. Protein complexes were isolated from the clarified cell lysate using 10μl anti-FLAG M2 affinity gel (Sigma-Aldrich) for 2h at 4°C. The beads were washed 7 times with 500μl of cell lysis buffer, wash buffer (50 mM Tris-HCl, pH 7.4, and 150mM NaCl, 0.1% [vol/vol] NP-40). Complexes were analyzed on 7.5%–10% SDS-PAGE gels or by western blotting.

#### Purification of longin family GEF complexes

To obtain Rab GEF complexes, FLAG and Myc-tagged forms of Intu and Fuzzy, Mon1 and Ccz1, or Hps1 and Hps4 were transiently expressed in 8x15cm dishes of 70% confluent HEK293T cells. For this purpose, 800μl OptiMEM (Invitrogen) was mixed with 24μl Mirus LT1, and after 5min 6μg of each plasmid DNA added. After 25min this transfection mix was added to the cells. After 40h growth the cell pellet was lysed for 20min on ice in 5ml cell lysis buffer (50mM Tris-HCl pH7.4, 1mM EDTA, 150mM NaCl, 0.5% [vol/vol] Triton X-100, protease inhibitor cocktail). Cell extracts were split into 1ml aliquots and clarified by centrifugation at 20,000xg in an Eppendorf 5417R microfuge for 20min. The FLAG-tagged proteins were isolated from the clarified cell lysate using 100μl anti-FLAG M2 affinity gel (Sigma) for 4h at 4°C. The beads were washed 7 times with 1ml of cell lysis buffer, 3 times with TBS and then the proteins were eluted with 100μl 200μg/ml FLAG-peptide in TBS containing 2mM dithiothreitol. Eluted proteins were analyzed on 7.5%–10% SDS-PAGE gels stained with Coomassie brilliant blue, and concentrations estimated by comparison to a series of bovine serum albumin standards in the range 0.1 to 1mg. The peak fractions were snap frozen in liquid nitrogen for storage at −80°C without dialysis.

#### Nucleotide binding and Rab GEF endpoint assays

Nucleotide binding and endpoint assays for GEF activity were carried out as follows [10, 23]. First, Rabs were loaded with nucleotide: 10μg GST-tagged Rab was incubated in 50 mM HEPES-NaOH pH 6.8, 0.1mg/ml BSA, 125μM EDTA, 10μM Mg-GDP, and 5μCi [^3^H]-GDP (10mCi/ml; 5000Ci/mmol) in a total volume of 200μl for 20min at 4°C. For standard GDP-releasing GEF assays 100μl of the loading reaction was mixed with 10μl 10mM Mg-GTP, 10-100nM GEF or a buffer control, and adjusted with assay buffer to 120μl final volume. The GEF reaction occurred for 20min at 30°C. After this, 2.5μl were taken for a specific activity measurement, the remainder was split into two tubes, then incubated with 500μl ice-cold assay buffer containing 1mM MgCl_2_, and 20μl packed glutathione-Sepharose for 60min at 4°C. After 3 washes with 500μl ice-cold assay buffer the Sepharose was transferred to a vial containing 4ml scintillation fluid and counted. The amount of nucleotide exchange was calculated in pmoles GDP-released. For GTP-binding assays the following modifications were made: only unlabelled GDP was used in the loading reaction; in the GEF reaction 0.5μl 10mM GTP and 1μCi [^35^S]-GTPγS (10mCi/ml; 5000Ci/mmol) were used. The amount of nucleotide exchange was calculated in pmoles GTP-bound.

#### Kinetic analysis of Rab GEF activity

For analysis of Rab GEF kinetics, 10nmol of hexahistidine-GST-Rab23 was loaded with 2′-(3′)-bis-*O*-(*N*-methylanthraniloyl)-GDP (Mant-GDP) (Jena Bioscience) in 20mM HEPES, pH 6.8, 1mg/ml BSA (protease and Ig-G free), 20mM EDTA, pH8.0, 40mM Mant-GDP at 30°C for 30min. After loading, 25mmol MgCl_2_ was added and the sample was exchanged into reaction buffer (20mM HEPES, pH6.8, 1mg/ml BSA (protease and Ig-G free), 150mM NaCl, 1mM MgCl_2_) using Zeba spin columns (Thermo Scientific). Nucleotide exchange was measured using 1nmol of the loaded Rab and the amount of GEF specified in the figure legends in a final volume of 100μl reaction buffer by monitoring the quenching of fluorescence after release of Mant-GDP using a Tristar LB 941 plate reader (Berthold Technologies) under control of MikroWin Software. Samples were excited at 350nm and emission monitored at 440nm. GTP was added to a final concentration of 0.1mM to start the exchange reaction at 30°C. Curve fitting and extraction of pseudo first order rate constants (k_obs_) was carried out using Microsoft Excel [23]. Since k_obs_ = (k_cat_/K_m_)x[GEF]+k_basal_ where k_basal_ is the rate constant measured in the absence of GEF, catalytic efficiency (k_cat_/K_m_) can be obtained.

#### Rab GTP-hydrolysis endpoint assays

For Rab-loading reactions, 10μl of assay buffer, 73μl H_2_O, 10μl 10 mM EDTA, pH 8.0, 5μl of 1 mM GTP, 2μl γ-[^32^P]GTP (10 mCi/ml; 5,000 Ci/mmol; ICN), and 100pmol Rab protein were mixed on ice. A 2.5 μL aliquot of the assay mix was scintillation counted to measure the specific activity in cpm/pmol GTP. Reactions were then incubated at 30°C for 60min. The 5 μL aliquots were immediately added to 795μl of ice-cold 5% [wt/vol] activated charcoal slurry in 50mM NaH_2_PO4, left for 1h on ice, and centrifuged at 16,100 g in a benchtop microfuge (5417R; Eppendorf) to pellet the charcoal. A 400μl aliquot of the supernatant was scintillation counted, and the amount of GTP hydrolysed was calculated from the specific activity of the reaction mixture.

#### Immunofluorescence microscopy of cilia in cultured cells

Cells were grown on No. 1.5 glass coverslips in 6-well plates. IMCD3 cells were plated at 30,000 cells per well and transfected with siRNA duplexes for 3 days. hTERT-RPE1 were plated at 20,000 cells per well and transfected with siRNA duplexes for 2 days or DNA for 1 day. To promote cilium formation, medium was replaced with growth medium lacking serum for 14h for IMCD3 or 48h for hTERT-RPE1 cells. For plasmid and siRNA transfection, Mirus TransIT-X2 (Mirus Bio LLC) and Oligofectamine (Invitrogen), respectively, were used according to the manufacturers’ instructions.

After the treatments described in the figure legends, cells were washed twice with 2ml of PBS at room temperature prior to fixation with either trichloracetic acid (TCA) or paraformaldehyde (PFA). For TCA-glycine fixation, cells were incubated in 2ml 10% [wt/vol] TCA at 4°C for 15min, and then washed five times in 2ml 30mM glycine, PBS pH7.4. For PFA fixation, cells were incubated in 2ml 3% [wt/vol] PFA in PBS for 15min, and then washed with 2ml 50mM ammonium chloride in PBS for 10min. Following fixation, all cells were permeabilised for 7min with 0.2% [vol/vol] Triton X-100 in PBS, followed by three washes in PBS. In all cases primary and secondary antibody staining was carried out in PBS for 60min at room temperature. Affinity purified antibodies were used at 1μg/ml while commercial antibodies were used as directed by the manufacturers. DAPI was added to the secondary antibody staining solution at 0.3μg/ml to stain DNA. Coverslips were mounted in Moviol 4-88 mounting medium (Calbiochem). Fixed samples on glass slides were imaged using a 60x NA1.35 oil immersion objective on an Olympus BX61 upright microscope with filtersets for DAPI, GFP/Alexa 488, Alexa-555, Alexa-568, and Alexa-647 (Chroma Technology Corp.), a CoolSNAP HQ2 camera (Roper Scientific), and Metamorph 7.5 imaging software (Molecular Dynamics Inc.). A Lumen 200 Watt metal halide light source (Prior Scientific Instruments Ltd) was used to illuminate the samples. Image stacks of up to 4 planes with a spacing of 0.3μm through the cell volume were taken. Image stacks were maximum intensity projected and then merged to create 24-bit RGB TIFF files in Metamorph. Images in 24-bit RGB format were then cropped in Photoshop CS3 and placed into Illustrator CS3 (Adobe Systems Inc.) to produce the figures.

#### Electron microscopy of cilia in cultured cells

For electron microscopy, hTERT-RPE1 cells were grown in 2cm dishes and treated as described in the legend to Figure 3. The cells were washed with 2ml PBS at room temperature, then fixed for 20min with 2.5% [vol/vol] glutaraldehyde in 0.1M sodium cacodylate buffer pH 7.4. Glutaraldehyde was removed by washing in 0.1M sodium cacodylate for 10min. Subsequently, cells were incubated for 30min with 1% [wt/vol] osmium tetroxide in 0.1M sodium cacodylate for 30min then washed 3x with water. Negative staining was performed for 20min with 3% [vol/vol] uranyl acetate. Prior to resin embedding, the cells were washed with water and dehydrated in a stepwise fashion by incubation in 70%, 80%, 90%, 96% and finally 100% ethanol for 10min per step. The cells were embedded in TAAB 812 resin (TAAB Laboratories Equipment Ltd, UK) and cured for 2 days in a 70°C oven. Thin 70nm sections were cut using a diamond knife then mounted on grids. Sections were post-stained on grids in 3% [wt/vol] uranyl acetate and lead citrate (Reynolds stain) and then examined with a FEI Tecnai12 transmission electron microscope.

#### Pupal wing dissection and imaging

Pupae were raised at 18°C, 25°C or 29°C as indicated and wings were dissected at the indicated time after puparium formation (APF). As *fy* mutants are cold sensitive, pupae were raised at 18°C for 64.5 hr for Actin labeling. Pupae expressing RNAi against *Rab23* under *GAL4-UAS* control were raised at 29°C to enhance the expression of dsRNA. Briefly, pupae were removed from their pupal case and fixed for 25-35 min in 4% [wt/vol] paraformaldehyde in PBS, depending on antibody combinations. Wings were then dissected and the outer cuticle removed, and were blocked for 1 hr in PBS containing 0.2% Triton X-100 (PTX) and 10% normal goat serum. Primary and secondary antibodies were incubated overnight at 4°C in PTX with 10% normal goat serum, and all washes were in PTX. Antibodies against Mwh (rat or rabbit) and Frtz (rabbit) have been described previously [34]. Western blotting of pupal wings used Mwh (affinity purified rabbit) and Actin (mouse monoclonal AC-40 #A4700; Sigma).

After immunolabelling, wings were post-fixed in 4% [wt/vol] paraformaldehyde in PBS for 30 min. Wings were mounted in 25 μl of PBS containing 10% [vol/vol] glycerol and 2.5% DABCO, pH7.5.

Pupal wings were imaged on a Leica SP1 confocal microscope using a 40x NA1.4 apochromatic lens or a Nikon A1R GaAsP confocal microscope using a 60x NA1.4 apochromatic lens. 9 Z-slices separated by 150 nm were imaged at a pixel size of 70-100 nm, and the 3 brightest slices around apicolateral junctions were selected and averaged for each channel in ImageJ.

#### Adult wing preparations

Adult wings were dehydrated in isopropanol and mounted in GMM (50% methyl salicylate, 50% Canada Balsam), and incubated overnight on a 60°C hot plate to clear. Wings were photographed at 20x magnification.

#### Western blotting of pupal wings

For pupal wing westerns, female larvae were raised at 19°C, and then shifted to 29°C for 27 hr APF before dissection of pupal wings directly into sample buffer. One pupal wing equivalent was used per lane.

### Quantification and Statistical Analysis

Details of the number of experimental repeats, numbers of cells analyzed and the relevant statistics are detailed in the figure legends. Data were plotted and statistical analysis performed using GraphPad Prism software.

#### Rab GEF and GAP endpoint assays

Nucleotide exchange and Rab GAP activity was measured in duplicate in 3 independent experiments. Mean values were plotted in bar graphs and error bars indicate the SEM. No statistical tests were performed.

#### Rab GEF kinetic assays

Nucleotide exchange over time was measured as a function of Rab GEF or GEF subunit concentration in 3 independent experiments. k_obs_ was obtained by fitting an exponential decay curve to the individual curves in Microsoft Excel. The mean k_obs_ value obtained was then plotted as a function of Rab GEF or GEF subunit concentration in GraphPad Prism. No statistical tests were performed.

#### Quantification of cilium formation and length

For cilium length, the freehand measuring tool of FIJI (ImageJ) was used to measure the length of individual cilia for 100 cells in each of 3 independent experiments. Acetylated tubulin and Arl13b were used as markers for this purpose. GraphPad Prism was used to calculate the mean length and SEM in μm for each experimental condition. These values were plotted in bar graphs.

To quantify the frequency of cilium formation, the number of elongated cilia and punctate ciliary vesicles were identified and counted using Arl13b staining as a marker for 100 cells in each of 3 independent experiments. GraphPad Prism was used to calculate the mean frequency and SEM for each experimental condition. These values were plotted in bar graphs. No statistical tests were performed.

### Data and Code Availability

This study did not generate or analyze datasets or code.

## Supplemental Information

Document S1. Figures S1–S4

Document S2. Article plus Supplemental Information
