# Supplementary material for: Planar Cell Polarity Effector Proteins Inturned and Fuzzy Form a Rab23 GEF Complex
Source: Curr Biol. 2019 Oct 7;29(19):3323–3330.e8. doi: 10.1016/j.cub.2019.07.090 (PMC6864590; doi:10.1016/j.cub.2019.07.090)
Supplement: Document S1. Figures S1–S4 [file mmc1.pdf]

Current Biology, Volume *29*

Supplemental Information

Planar Cell Polarity Effector Proteins Inturned and Fuzzy Form a Rab23 GEF Complex

Andreas Gerondopoulos, Helen Strutt, Nicola L. Stevenson, Tomoaki Sobajima, Tim P. Levine, David J. Stephens, David Strutt, and Francis A. Barr

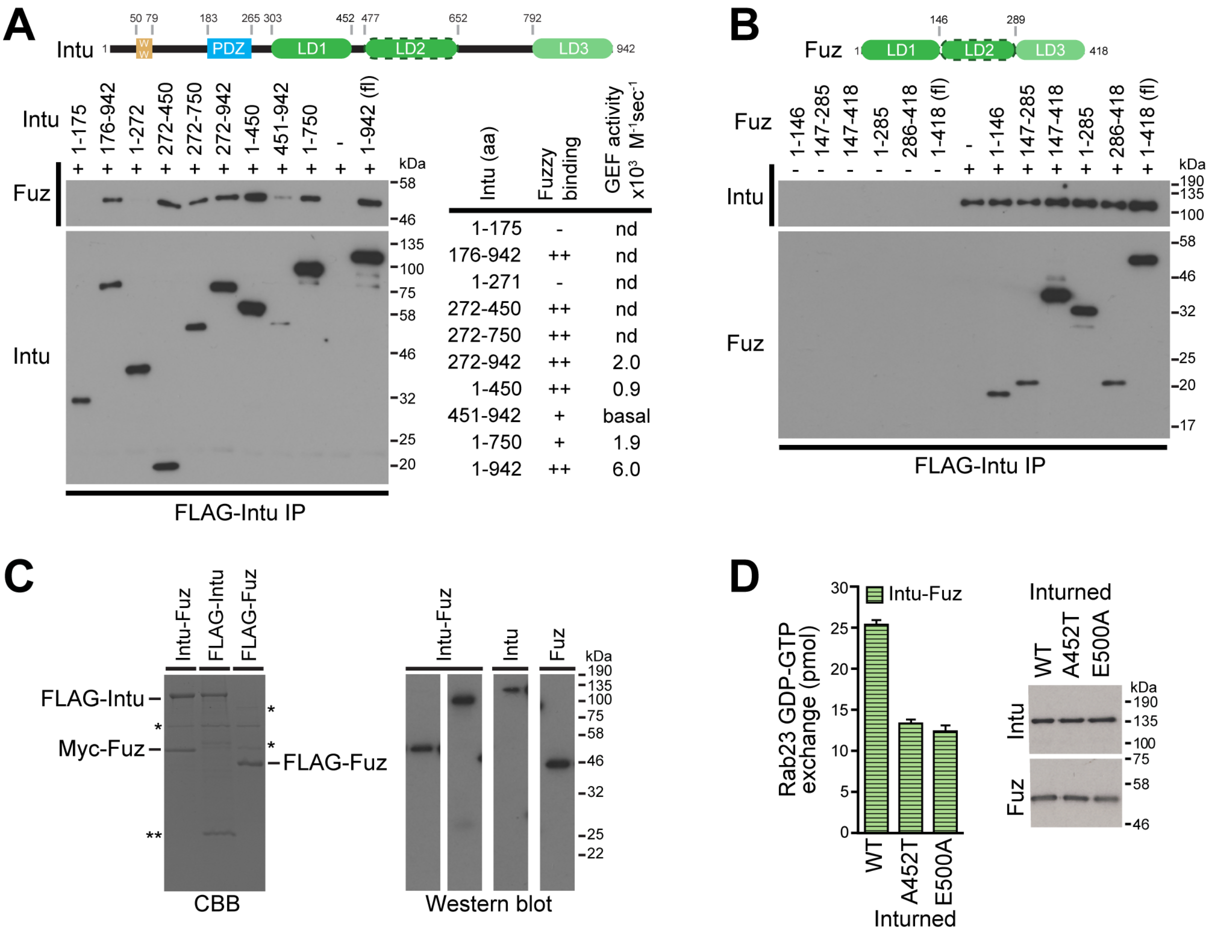

Figure S1. Characterisation of the Intu-Fuz complex. Related to Figure 1.

(A) Predicted domain structure of human Intu. HEK293T cells were co-transfected with FLAG-Intu and myc-Fuz constructs and after 24h complexes were recovered by FLAG IP. Fuz binding properties of Intu deletion constructs (scored from negative (-) to positive (++) and Rab23 GEF activity are summarized in the inset table. **(B)** Predicted domain structure of human Fuz. HEK293T cells were transfected with myc-Fuz or FLAG-Intu and myc-Fuz constructs for 24h. Complexes were recovered using FLAG IP. **(C)** Intu and Fuz complexes and individual subunits were analyzed on Coomassie brilliant blue (CBB) stained protein gels and by western blot to confirm the presence of the individual subunits. Asterisks mark contaminant proteins. **(D)** Rab23 GEF activity of Intu-Fuz complexes containing WT or disease associated mutant forms of Intu. Error bars indicate the SEM (n=2 independent conditions).

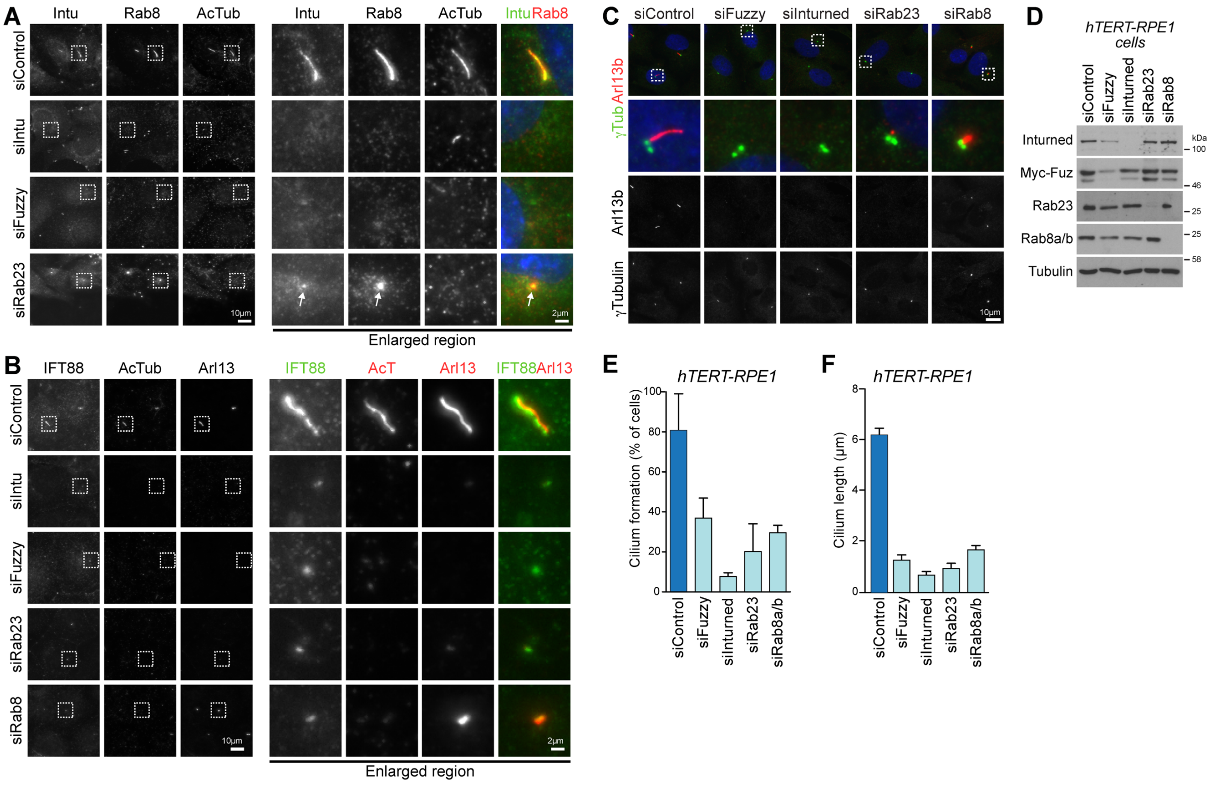

Figure S2. Rab23 GEF localises to cilia and is required for cilium formation. Related to Figure 2.

(A) IMCD3 cells were depleted of Intu, Fuz or Rab23 for 72h and then induced to form cilia for 14h. The cells were fixed with TCA-glycine and then stained with antibodies to Inturned, Rab8 and acetylated tubulin (AcTub) or **(B)** IFT88, Arl13b and acetylated tubulin. **(C)** hTERT-RPE1 cells expressing myc-tagged Fuz were depleted of Intu, Fuz, Rab23, or Rab8 for 48h then and then induced to form cilia for a further 48h. Cells were fixed with TCA-glycine and then stained with antibodies for Arl13b and γ-tubulin to determine the extent of cilium formation or **(D)** analysed by western blotting to confirm depletion of target proteins. **(E)** Cilium formation (100 cells per condition in 3 independent experiments) and **(F)** cilium length (40 cells per condition in 3 independent experiments) were measured using Arl13b. Errors bars indicate the SEM.

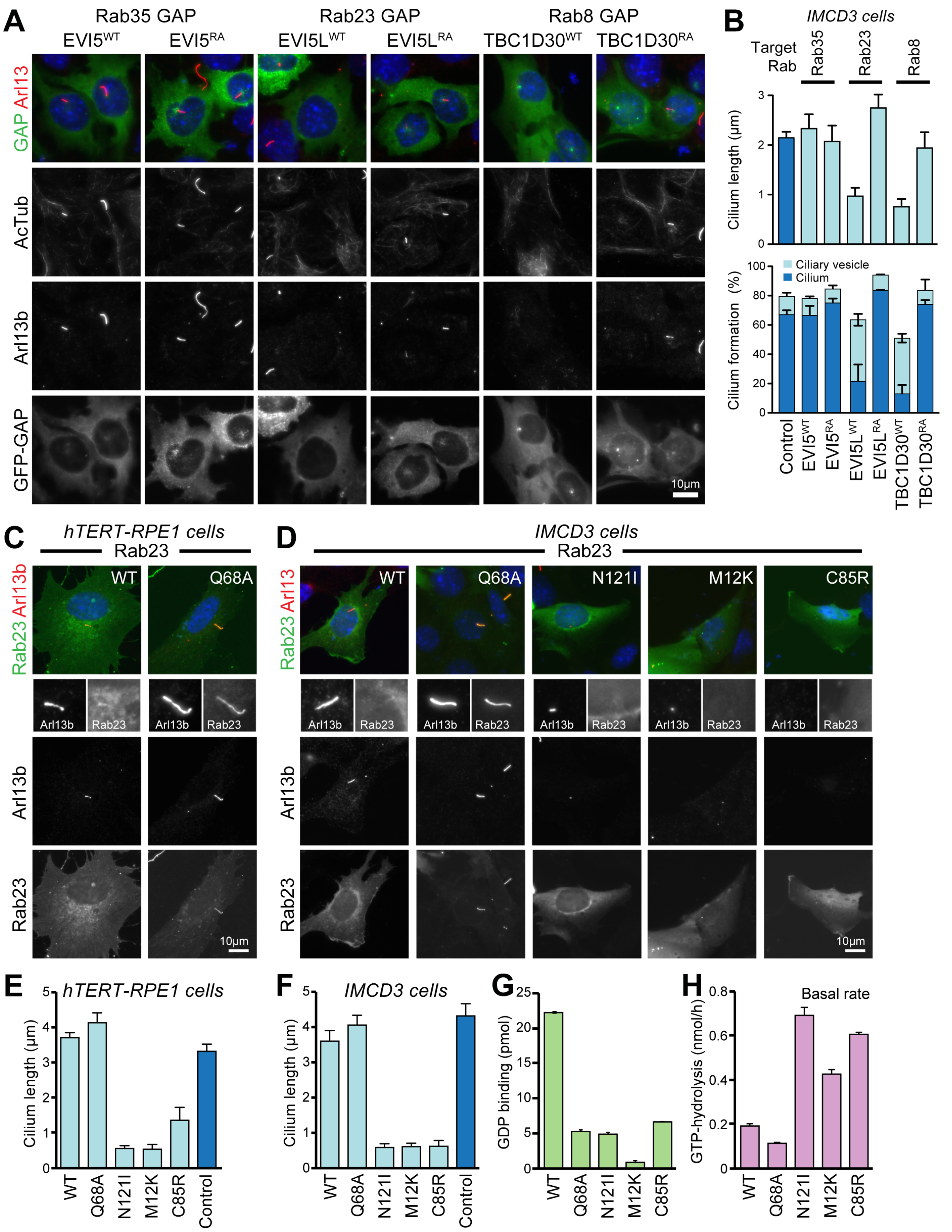

Figure S3. Specific Rab GAPs inhibit cilium formation in IMCD3 cells. Related to Figure 2.

(A) IMCD3 cells were transfected for 24h with WT or catalytically inactive (RA) GFP-tagged RabGAPs acting on Rabs implicated in cilium function. Cells were then

serum starved to induce cilium formation. After a further 48h the cells were fixed with PFA and then stained for acetylated tubulin (AcTub) and Arl13b as markers for primary cilia. DNA was stained with DAPI. Bars, 10 µm. **(B)** Elongated cilia and punctate ciliary vesicles were identified using Arl13b staining (100 cells per condition in 3 independent experiments). Cilium length was measured using Arl13b as a marker in both RabGAP expressing and non-expressing (control) cells for 50 cells in each of 2 independent experiments. The mean cilium length for the non-transfected cells in all conditions is plotted as the control. Error bars are the SEM. **(C)** Human telomerase immortalised retinal pigment epithelium (hTERT-RPE1) or **(D)** mouse inner medullary duct collecting cells were transfected with wild-type (WT), dominant active (Q68A), dominant negative (N121I) and Carpenter syndrome associated mutant (M12K and C85R) versions of GFP-Rab23. Serum starvation was used to induce cilium formation. After 48h the cells were fixed with PFA and then stained for Arl13b and DNA (blue). Enlarged insets show the details of the cilia. **(E and F)** Cilium length, defined by Arl13b staining, was measured in non-transfected cells (control) and cells expressing WT or mutant Rab23 for 30 cilia. Error bars indicate the SEM for 3 independent experiments. **(G)** GDP-binding and **(H)** basal GTP-hydrolysis were measured for WT and mutant Rab23, error bars indicate the SEM for 3 independent experiments.

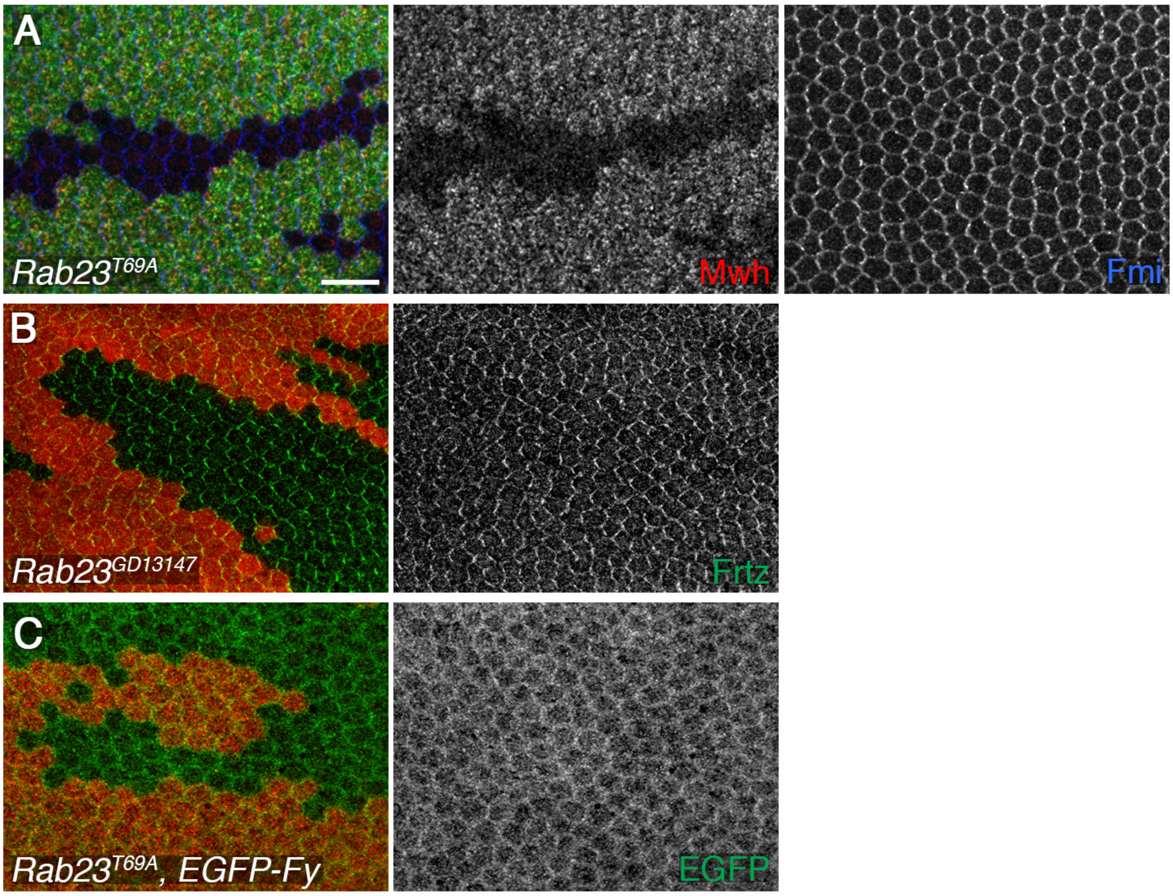

Figure S4. Effects of Rab23 on Mwh and Rab23 GEF localisation. Related to Figure 4.

(A) 32 hr APF pupal wing carrying loss of functions clones of *Rab23* marked by loss of ß-gal labelling (red) and immunolabelled for Mwh (red) and Fmi (blue). **(B)** Pupal wing with clones expressing RNAi against *Rab23*, marked by ß-gal labelling (red)**.** Pupae raised at 29°C for 25h and wings immunolabelled for Fritz (green). **(C)** 32 hr APF pupal wing expressing *EGFP-Fy* and carrying loss of functions clones of *Rab23* marked by loss of ß-gal labelling (red). Wings immunolabelled for GFP (green). Scale bar is 10 µm.
